# Obesity and pre-hypertension in family medicine: Implications for quality improvement

**DOI:** 10.1186/1472-6963-7-212

**Published:** 2007-12-21

**Authors:** James E Rohrer, Gregory J Anderson, Joseph W Furst

**Affiliations:** 1 Department of Family Medicine, Mayo Clinic, Rochester, Minnesota USA

## Abstract

**Background.:**

Prevention of pre-hypertension is an important goal for primary care patients. Obesity is a risk factor for hypertension, but has not been addressed for pre-hypertension in primary care populations. The objective of this study was to assess the degree to which obesity independently is associated with risk for pre-hypertension in family medicine patients.

**Methods.:**

This study was a retrospective analysis of information abstracted from medical records of 707 adult patients. Multivariable logistic regression was used to test the relationship between body mass index (BMI) and pre-hypertension, after adjustment for comorbidity and demographic characteristics. Pre-hypertension was defined as systolic pressure between 120 and 139 mm Hg or diastolic pressure between 80 and 89 mm Hg.

**Results.:**

In our sample, 42.9% of patients were pre-hypertensive. Logistic regression analysis revealed that, in comparison to patients with normal body mass, patients with BMI > 35 had higher adjusted odds of being pre-hypertensive (OR = 4.5, CI 2.55–8.11, p < .01). BMI between 30 and 35 also was significant (OR = 2.7, CI 1.61–4.63, p < 0.01) as was overweight (OR = 1.8, CI 1.14–2.92, p = 0.01).

**Conclusion.:**

In our sample of family medicine patients, elevated BMI is a risk factor for pre-hypertension, especially BMI > 35. This relationship appears to be independent of age, gender, marital status and comorbidity. Weight loss intervention for obese patients, including patient education or referral to weight loss programs, might be effective for prevention of pre-hypertension and thus should be considered as a potential quality indicator.

## Background

Obesity is an independent risk factor for a number of chronic diseases, including hypertension, diabetes, knee replacement, pancreatitis, insomnia, chronic fatigue, and early death[1]. It also is related to pain arising from arthritis, migraine progression, orthopedic disorders and general pain[2-4]. It may also be a risk factor for borderline conditions such as pre-hypertension. Since the aging of the population is expected to increase the prevalence of chronic diseases in the population, thus creating strains on the medical care system, modifications of clinical practice that are designed to reduce risk factors are of increasing importance[5]. Body weight in obese patients is an important example of a modifiable risk factor.

Weight loss might aid in prevention of pre-hypertension. However, the relevance of weight loss interventions as quality indicators has not been explored. This is an interesting omission, since action to encourage another type of behavior change, smoking cessation, is regarded as good medical care.

The objective of this study was to assess the degree to which obesity independently is associated with risk for pre-hypertension in family medicine patients. If obesity increases risk to a clinically relevant extent even after adjustment for age, comorbidity and gender, then in-clinic counseling or referral to weight management programs might be indicated. We use these findings as a starting point to discuss obesity as a quality indicator and potential barriers and strategies for effecting weight loss in primary care clinics.

## Methods

A random sample of adult patients treated in our clinics (five clinics, all in the Rochester area) and referred for specialist consultations was obtained to evaluate specialty referrals in our department. The data were reanalyzed for the present study. The sample was drawn during the period January through June of 2006. During the period June 2006 through March 2007, patients for whom virtual specialist visits were ordered were added to the data base. An index visit was defined as the visit in which a specialist consultation was ordered. Medical records were the sole source of information used in this study.

Blood pressure was recorded for 989 adult patients at the index visit. BMI was missing for 147 patients. Patients whose blood pressure exceeded 140/90 mm Hg were excluded since the research question concerned pre-hypertension. Our final sample included 707 subjects for whom systolic and diastolic pressures, severity, body mass index, age, gender and marital status information was available within six months before or after the index date. Data collection was approved by the local Institutional Review Board as a component of an evaluation of specialty consultation visits. The requirement for obtaining patient consent was waived by the IRB since this was a retrospective review of medical records limited to patients who had granted blanket approval for such studies.

The dependent variable in this study was pre-hypertension (yes or no), defined as systolic pressure between 120 and 139 mm Hg or diastolic pressure between 80 and 89 mm Hg. Single blood pressure measurements were used in order to minimize missing data.

Independent variables included body mass index (BMI), age, gender, marital status, and severity of illness. Nurses measured weight and height as a routine component of clinic visits and our electronic medical record automatically computed body mass index (BMI). BMI was categorized as "normal" if below 25, "overweight" if between 25 and 30, "Obese I" if between 30 and 35, and "Obese II" if over 35.

Age was broken into categories: 18–35, 36–45, 46–55, 56–65, and 66–100. Marital status was coded as married or not married.

The Charlson index was used to score co-morbidity[6]. Two trained medical secretaries were used as abstractors. Abstractors searched online medical records to locate diagnoses used in the Charlson system, whether they were recorded in billing codes or in narrative form in the problem list. The Charlson system was modified as follows. First, each case was scored using the following points: Peripheral vascular disease (1), Lymphoma multiple myeloma (2), Leukemia (2), Metastatic tumor (6), Other cancer (2), Connective tissue disease (1), Myocardial Infarction (1), Congestive Heart Failure (1), Cerebrovascular Disease (1), Chronic Pulmonary Disease (1), Dementia (1), Diabetes (1), Moderate Renal Disease (1), Severe Renal Disease (2), Ulcer disease (1), Mild liver disease (2), Other liver (3), Paralysis (1), AIDS (6). Second, the scale was collapsed into three categories: zero comorbidity (zero points), low comorbidity (1 point), and moderate comorbidity (2 or more).

Reliability checks were performed to assure coding was being performed in the same way by both raters. Blinded agreement between raters on the total severity score was 80 percent.

Independent variables, including BMI categories, were compared to hypertensive status in univariate tests using chi-square. A difference between categories was considered statistically significant if *p *< 0.05. We then performed multivariable logistic regression analyses to identify independent associations between the various independent variables and the odds of being pre-hypertensive. All demographic and clinically relevant variables were forced into the model. These included BMI, Charlson score, age, gender, and marital status. Statistical analysis was performed using EpiInfo version 3.3.2.

## Results

Table 1 reports sample demographics for the independent variables. The typical respondent was a married female over 45 years of age. Most had BMIs over 25. Over 60 percent were scored in the lowest severity category. The percentage of patients who were pre-hypertensive increased with BMI (p < .01), age (p < .01) and comorbidity (p < .01). Males were more likely to be pre-hypertensive than females (p < .01). Marital status was unrelated to being pre-hypertensive (p = .15).

**Table 1:** Descriptive Statistics: Independent Variables (N = 707)

	**Percent Pre-hypertensive (N = 303)**	**Percent Not Pre-hypertensive (N = 404)**	**Total Percents**	**Odds Ratio**	**N**
BMI (p < .001)					
Normal	15.2	33.9	25.9	1.00	183
Overweight	28.1	26.7	27.3	2.48	193
Obese I	22.4	13.1	17.1	3.55	121
Obese II	18.8	8.4	12.9	4.52	91
Missing	15.5	17.8	16.8	1.76	119
Age (p < .001)					
18–35	11.9	38.4	27.0	1.00	191
36–45	16.2	15.6	15.8	3.10	112
46–55	20.1	20.0	20.1	3.50	142
56–65	16.8	11.1	13.6	5.42	96
66–100	35.0	14.9	23.5	7.86	166
Gender (p = .002)					
Female	56.4	67.6	62.8	1.00	444
Male	43.6	32.4	37.2	1.74	263
Married (p = .149)					
No	24.1	29.0	26.9	1.00	190
Yes	75.9	71.0	73.1	1.34	517
Comorbidity (p = .003)					
None	55.1	67.6	62.2	1.00	440
Low	23.1	17.1	19.7	1.55	139
Moderate	21.8	15.3	18.1	1.78	128

Multivariable logistic regression analysis was performed to determine the independent effects of BMI category on pressure (see Table 2). Results confirmed the univariate analysis. Overweight (adjusted odds ratio = 1.82, CI (confidence interval 1.14–2.92, p = .01), obese I (OR = 2.73, CI 1.61–4.63, p < .01), and obese II (OR = 4.55, CI 2.55–8.11, p < .01) were related to increased odds of being pre-hypertensive. Risk increased with age. Gender, marital status and comorbidity were not significantly related to pre-hypertensive status after adjusting for BMI.

**Table 2:** Multivariable Logistic Regression Analysis of Pre-Hypertension Risk (N = 707)

**Variable**	**Odds Ratio**	**Lower Confidence Limit**	**Upper Confidence Limit**	**P**
Comorbidity				
None	1.00			
Low	1.12	0.72	1.73	0.62
Moderate	0.76	0.47	1.22	0.26
Age group				
18–35	1.00			
36–45	2.88	1.66	5.01	0.00
46–55	2.79	1.63	4.78	0.00
56–65	4.50	2.48	8.17	0.00
66–100	7.75	4.49	13.38	0.00
Male (vs. female)	1.36	0.97	1.91	0.08
Married (vs unmarried)	0.83	0.55	1.23	0.35
BMI category				
Normal	1.00			
Overweight	1.82	1.14	2.92	0.01
Obese I	2.73	1.61	4.63	0.00
Obese II	4.55	2.55	8.11	0.00
Missing	2.14	1.26	3.64	0.01

## Discussion

The relationship between obesity and hypertension in the general population has been reported [7]. Greenlund et al demonstrated a relationship between pre-hypertension and both overweight and obesity using data from the National Health and Nutrition Examination Survey, concluding that early clinical detection of pre-hypertension and intervention, including lifestyle modification programs, are needed[8]. Our study differs in that we focus on obesity in a primary care population. Furthermore, in contrast to earlier investigations, we are led to the conclusion that early intervention could be triggered by elevated BMI instead of waiting for blood pressures to become elevated. In addition to intensive comprehensive lifestyle modification programs targeted at hypertension[9,10], we see the value of weight management programs.

Both prevention of pre-hypertension and treatment of it are worthwhile. The JNC 7[11] defined pre-hypertension as 120–139 mm Hg (systolic) or 80 to 89 mm Hg (diastolic) and recommended lifestyle changes including weight loss for patients in this category. The U.S. Preventive Services Task Force (USPSTF) concluded that the evidence was insufficient to recommend for or against routine behavioral counseling to promote a healthy diet in unselected patients in primary care settings. However, intensive behavioral dietary counseling was recommended for adult patients with known risk factors for cardiovascular and diet-related chronic disease. Also described by the USPSTF as 'promising' were medium-intensity counseling and lower-intensity counseling. Medium-intensity counseling, according to the USPSTF, involves two to three visits with specially trained primary care clinicians or by referral to nutritionists or dietitians [12]. Lower-intensity interventions involving 5 minutes or less of primary care provider counseling supplemented by patient self-help materials, telephone counseling or interactive health communications.

Intensive dietary counseling is costly and time-consuming and, possibly, no more effective than referring patients to commercial weight loss programs. Until insurance companies begin reimbursement for weight loss counseling, primary care clinics will need to look for less expensive alternatives. Even medium-intensity counseling may be impractical at this juncture. Lower-intensity counseling, on the other hand, might trigger patient participation in self-help or commercial weight loss systems. As with cigarette smoking, re-starts may be required before success is achieved.

In our patients, having BMI > 35 more than quadrupled the odds of having pre-hypertension. Previous work has shown that weight loss can be effective in reducing blood pressure in this group, alone or in combination with other lifestyle changes[13,14]. We infer that active intervention to reduce body weight for patients who are morbidly obese and have elevated blood pressure is obligatory, with due respect for patient sensitivities about their body weight. Weight management interventions for patients with BMI > 35 also is valuable before blood pressure becomes elevated or other cardiac risk factors develop. Patient education or, possibly, referral to weight loss programs should be considered[15]. Scheduling a visit for patient education is more costly than referral to an external weight loss program and may not be any more effective.

About 17 percent of the patients in our sample for whom blood pressure was recorded were missing recent BMIs. Of those for whom complete information was available, approximately 20 percent were obese. Over half of obese patients were pre-hypertensive. Modest weight loss in obese patiens could lower blood pressures into a safer range. We suspect that offering weight loss services to these patients would identify motivated volunteers, most of whom would successfully lose sufficient weight to drop down one BMI category. Elevations in pressures due to clinical problems that are independent of obesity would remain unaffected. Nevertheless, improvements in blood pressure should be possible in many patients. Financing remains an issue, however, since many insurance companies do not cover weight management services.

Our findings may aid in clarifying recommendations for weight management in primary care. Many primary care practices have no standard of care concerning either patient education or referral of obese patients to weight loss programs, though these would be useful[15-18]. Since many primary care patients are ready to lose weight, timely advice[18] and referrals to weight loss programs could help them make significant changes.

Classification as 'pre-hypertensive,' or even 'at risk' for pre-hypertension, may cause obese patients to take notice, thus creating a 'teachable moment.' That is the point where providers may exert more leverage for moving patients into medium intensity counseling. This intervention point can be described as primary prevention and as such may offer an earlier, lower-cost approach than waiting until the patient is diagnosed with hypertension. Motivational Interviewing is one approach for introducing brief counseling into primary care settings [19]. Few studies have been reported demonstrating its effectiveness for weight loss, but research projects are in progress and some reports are beginning to appear [20].

Many clinicians discuss lifestyle modification with patients in the pre-hypertensive category. As much as BMI is a reflection of lifestyle, addressing it would be appropriate when patients are found to be in that range. Associating elevated BMI with blood pressures above the optimal range reinforces the association of obesity as a risk factor for hypertension.

As a practical matter, it can be difficult at times to know if blood pressures obtained in the office reflect the patient's blood pressure in other settings. The significance of a blood pressure reading in the pre-hypertensive range may therefore be unclear. An elevated BMI in that situation, being associated with pre-hypertension, may suggest that the patient is indeed at increased risk of progressing to frank hypertension Early intervention through lifestyle modification may lessen the risk of progression.

Our findings are consistent with other studies [21]. However, resistance to recommendations about taking action on pre-hypertension has emerged and both sides have been articulately expressed[21-23]. Providers may believe that counseling and other weight loss programs do not work[23,24]. However, a variety of approaches can be effective if the patient is ready for change, including commercial programs. The BBC study, for example, was a rigorous trial comparing several weight loss plans. All were somewhat effective [25]. Of course, all subjects were volunteers, indicating some readiness for lifestyle change. This explains why providing counseling to all overweight patients seen in clinics is ineffective while at the same time counseling patients who seek assistance can achieve dramatic results.

This study relies on cross-sectional data to test hypotheses, thus we cannot draw conclusions about causal relationships. Another limitation of our study is that the sample was potentially not representative of either the community or the family medicine patients seen in our clinics. Finally we note that single blood pressure measures were used in the manuscript, introducing the possibility of regression dilution bias. Since blood pressures frequently are not recorded, we minimized loss of cases by making this decision.

## Conclusion

Since the JNC 7 reclassification of hypertension into normal, pre-hypertension and hypertension stages 1 and 2, more attention has been paid to patients with blood pressure above optimal but previously considered to be normal. Our findings demonstrate an association between progressively increasing BMI and progressively increasing risk of pre-hypertension.

Obesity is common in primary care patients, including those with pre-hypertension. Focusing on medication adjustments may cause primary care providers to overlook the possibility of non-medical strategies, such as weight loss programs. We propose that two new quality indicators be considered: 1) BMIs should be recorded in the medical record at least biannually for all patients who visit their providers biannually; and 2) for morbidly obese patients, documentation should be found in the record indicating that some action was taken, such as patient education or referral to a weight loss program. At a minimum, educational materials should be offered that will allow patients to pursue self-help strategies. The purpose of this second recommendation is to prevent pre-hypertension and other medical consequences of obesity.

Routine recording of BMI in the electronic record is an important prerequisite to increasing referrals to weight management programs [26]. Consensus on the ideal type of weight management program is elusive. This is an evolving field and providers may need periodic updates about the newest evidence on the relative effectiveness of different programs. At this juncture, offering patients a menu of options and urging them to choose the most appealing one might be the most appropriate action.

Additional research is needed to test the relative effectiveness of different approaches to weight loss. Variables include the number and length of visits required to help patients lose weight, the types of providers who can most cost-effectively provide the service (nurses, health educators, or counselors), and the best modalities (internet, group classes, or personal health services). Since we lack firm evidence on the merits of different approaches, the decision about what type of service should be recommended can be left to patient preferences.

## Competing interests

The author(s) declare that they have no competing interests.

## Authors' contributions

JER designed the study, analyzed the data and wrote the first draft. GA and JF wrote additional material. All authors have read and approved the final version of the manuscript.

## Pre-publication history

The pre-publication history for this paper can be accessed here:


